# Targeting Non-Coding RNAs as a Potential Therapeutic and Delivery Strategy Against Neurodegenerative Diseases

**DOI:** 10.3390/ijms27073260

**Published:** 2026-04-03

**Authors:** Anastasia Bougea

**Affiliations:** Medical School, National and Kapodistrian University of Athens, M. Asias 75, 11574 Athens, Greece; abougea@med.uoa.gr

**Keywords:** non-coding RNAs, neurodegenerative diseases, nanotechnology, ncRNA, CRISPR/Cas13, RNA therapeutics, precision medicine, exosomes, gene regulation, blood–brain barrier

## Abstract

Neurodegenerative diseases (NDs), including Alzheimer’s disease, Parkinson’s disease, Huntington’s disease, and amyotrophic lateral sclerosis (ALS), represent a growing global health challenge characterized by progressive neuronal loss and a lack of definitive disease-modifying treatments. This review explores the emerging potential of targeting non-coding RNAs (ncRNAs), such as microRNAs (miRNAs), long non-coding RNAs (lncRNAs), and exosomal RNAs, to modulate pathogenic molecular pathways and address the underlying molecular origins of neurodegeneration. We evaluate the integration of advanced computational techniques for RNA structure prediction and gene regulatory network analysis, alongside chemical engineering strategies—such as Locked Nucleic Acids (LNAs) and phosphorothioate modifications—aimed at enhancing the stability and specificity of RNA-based molecules. Furthermore, we analyze cutting-edge delivery and editing technologies, including nanotechnology-driven solutions for precise neuronal targeting and the CRISPR/Cas13 system for direct ncRNA manipulation.The findings indicate that while challenges in delivery efficiency and long-term efficacy persist, the synergy of chemical engineering and computational modeling significantly improves the therapeutic profile of ncRNAs, with exosomal pathways offering a novel route for intercellular signaling modulation and biomarker discovery. Therapeutic interventions directed at specific clinical targets, such as miR-34a and BACE1-AS, demonstrate the capacity to influence protein aggregation and neuroinflammatory cascades. Although ncRNA-based therapies are currently in nascent stages, ongoing technological advancements in RNA editing and nanotechnology offer a transformative framework that could redefine the future of ND treatment and successfully halt disease progression rather than merely managing symptoms.

## 1. Introduction

Neurodegenerative diseases (NDs) are characterized by the progressive degeneration and death of neurons in specific parts of the nervous system [1]. This neurodegeneration ultimately manifests as severe cognitive impairment, motor dysfunction, and diverse neurological deficits in conditions such as Alzheimer’s Disease (AD), Parkinson’s Disease (PD), Huntington’s Disease (HD), and Amyotrophic Lateral Sclerosis (ALS) [2,3,4,5,6]. Historically, conventional pharmacological strategies have focused heavily on targeting downstream transcriptional or translational pathways—primarily relying on small molecules to modulate protein function [7]. However, these traditional therapies have largely failed to modify the underlying disease progression, leaving many neurodegenerative conditions classified as previously “untreatable” [8].

This persistent clinical failure has driven a critical paradigm shift toward RNA-based treatments, which present a potent alternative by controlling gene expression at the foundational molecular level [3]. Rather than targeting synthesized proteins, RNA therapeutics—which include messenger RNA (mRNA), small interfering RNA (siRNA), antisense oligonucleotides (ASOs), and microRNAs (miRNAs)—intervene directly at the post-transcriptional stage [4]. This approach offers distinct clinical advantages: it allows for the precise and selective targeting of specific genetic defects, the delivery of synthetic genes to generate therapeutic proteins [5], and the manipulation of gene expression without permanently altering the host’s DNA code by binding to complementary mRNA sequences [6,8].

Central to this new therapeutic frontier are non-coding RNAs (ncRNAs). While these RNA molecules do not translate into proteins, they serve as master regulators of cellular functions and gene expression networks [9,10]. The most extensively studied classes—microRNAs, long non-coding RNAs, and circular RNAs [11]—are deeply involved in the regulation of RNA splicing, stability, and translation [10]. In the context of NDs, pathogenesis is frequently driven by the overexpression or underexpression of specific gene networks; modulating these regulatory functions using precise ncRNA tools (such as hybridizing short DNA-like ASOs to target RNAs) has emerged as a highly promising therapeutic strategy to restore cellular homeostasis [12,13,14].

Despite their vast potential, the clinical translation of RNA therapeutics faces formidable biological limitations. Naked RNA is inherently unstable, and delivering these macromolecules across the highly selective blood–brain barrier (BBB) into central nervous system tissues remains a significant challenge [15,16,17,18,19]. Therefore, identifying a molecular target is only the first step; successful intervention requires a multidisciplinary approach [15,16,17,18,19,20,21].

In modern ND research, combining computational and chemical approaches plays a crucial role in accelerating drug discovery [15]. Advanced computational techniques, including Machine Learning (ML) algorithms, are essential for analyzing massive RNA expression datasets to uncover disease-specific alterations in ncRNA expression and optimize drug specificity. Methodologically, this involves mapping comprehensive catalogs of ncRNAs and utilizing target prediction algorithms to evaluate base-pairing interactions between miRNA sequences and the 3′ untranslated region (UTR) of target mRNAs [17]. Furthermore, robust in silico predictive modeling of secondary and tertiary RNA structures enables researchers to accurately map functional domains. This structural modeling is vital for guiding the rational design of highly specific therapeutic molecules, including miRNA mimics, inhibitors, and ASOs [16].

Concurrently, chemical engineering is required to translate these computational predictions into viable clinical therapies. The bioavailability and stability of RNA interventions are vastly improved through chemical modifications of the oligonucleotides themselves, alongside the rational design of advanced delivery systems capable of traversing the BBB [18]. Ultimately, combining computational target prediction—including in silico molecular docking and quantitative structure-activity relationship (QSAR) models —with chemical stabilization strategies permits the design of highly targeted, precise, and effective RNA-based treatments [19].

Ultimately, combining computational target prediction with chemical stabilization strategies permits the design of highly targeted, precise, and effective RNA-based treatments [20]. The primary aim of this review is to critically evaluate the role of ncRNAs in neurodegeneration and explore how the synergistic application of computational modeling and chemical engineering can overcome existing delivery barriers, maximizing the probability of developing personalized, disease-modifying therapies that target NDs at their molecular roots [15,20,22,23,24,25].

## 2. Molecular Pathogenesis and Target Networks

### 2.1. miRNAs: Master Regulators of Neurodegeneration ncRNA Dysregulation in ND Pathogenesis

**Figure 1:**
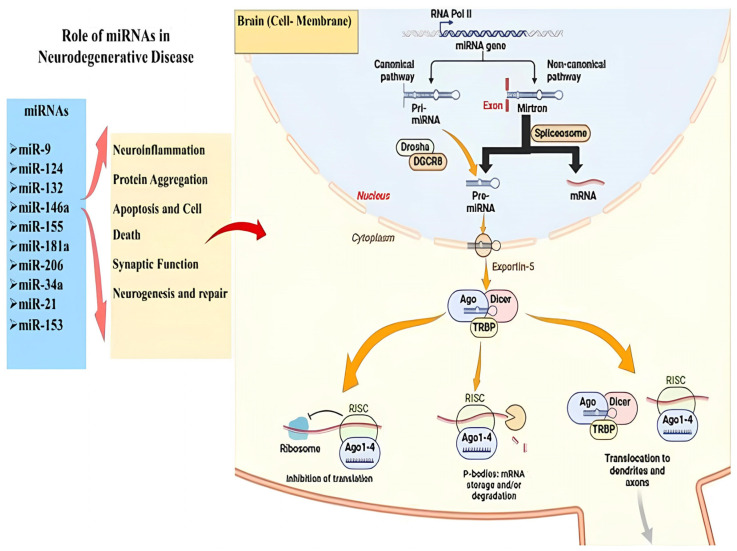
The role of miRNA in regulating gene expression and preserving neuronal function. Mature miRNAs within the RISC target specific mRNAs for translational repression or degradation, regulating functions such as synaptic plasticity and neurodevelopment.

In the central nervous system, non-coding RNAs (ncRNAs) act as master regulators of cellular functions, simultaneously controlling gene networks that govern neurogenesis, synaptic plasticity, and apoptosis [26,27]. Specific miRNAs, typically 18 to 24 nucleotides in length, act post-transcriptionally by inducing the degradation or translational repression of target mRNAs [26,27]. Figure 1 illustrates the intricate intracellular biogenesis of miRNAs and highlights their master regulatory roles in neurodegeneration. The canonical maturation process begins in the nucleus, where RNA polymerase II transcribes miRNA genes into primary miRNAs (pri-miRNAs), which are subsequently cleaved by the Drosha/DGCR8 microprocessor complex. Non-canonical pathways, such as spliceosome-dependent mirtrons, also contribute to the pre-miRNA pool. Following Exportin-5-mediated translocation into the cytoplasm, the pre-miRNA is further processed by the Dicer/TRBP complex and loaded onto Argonaute (Ago) proteins to form the active RNA-induced silencing complex (RISC). In the context of the central nervous system, this RISC dynamically regulates specific neurodegenerative networks—including neuroinflammation (e.g., miR-146a, miR-155) and protein aggregation (e.g., miR-9, miR-124)—by either inhibiting mRNA translation at the ribosome, directing transcripts to P-bodies for degradation, or translocating to axons and dendrites to regulate local synaptic function.

Concurrently, long non-coding RNAs (lncRNAs), defined as transcripts exceeding 200 nucleotides, act as critical scaffolds and guides that dictate how RNA-binding proteins and chromatin modifiers interact with cellular machinery to modulate gene expression [28,29,30,31,32]. This overarching “epigenetic symphony” is further modulated by histone acetylation, which alters chromatin accessibility at specific loci, and complex epitranscriptomic RNA modifications that dictate the structural stability and precise localization of these transcripts [32]. Consequently, when these interconnected regulatory networks become dysregulated, they disrupt protein homeostasis, exacerbate neuroinflammation, and accelerate neuronal cell death, functioning as a principal pathogenic determinant across neurodegenerative diseases [33,34].

#### 2.1.1. Protein Aggregation and Pathological Propagation

Neurodegeneration is heavily characterized by the accumulation of misfolded proteins, such as amyloid-beta in Alzheimer’s Disease (AD) and α-synuclein in Parkinson’s Disease (PD) [35]. In AD, the molecular cascade is driven by the lncRNA BACE1-AS, which specifically binds to the BACE1 transcript, preventing its degradation and thereby upregulating the beta-secretase enzyme responsible for the pathogenic cleavage of amyloid precursor protein (APP) into toxic oligomers [36]. Additionally, exosomal miR-9, miR-132, and miR-146a modulate APP expression, directly influencing amyloid-beta accumulation [37], while the overall impairment of these crucial miRNAs compromises the degradation and clearance of these toxic proteins [38,39,40]. In PD, pathogenesis is intrinsically linked to the *SNCA* gene; specific miRNAs normally repress *SNCA* expression, and their age-related downregulation leads to the toxic accumulation and aggregation of alpha-synuclein into Lewy bodies. Similarly, lncRNAs are heavily implicated in the aggregation of mutant huntingtin (mHTT) proteins in HD and the dysregulation of motor neuron survival pathways in ALS.

**Figure 2:**
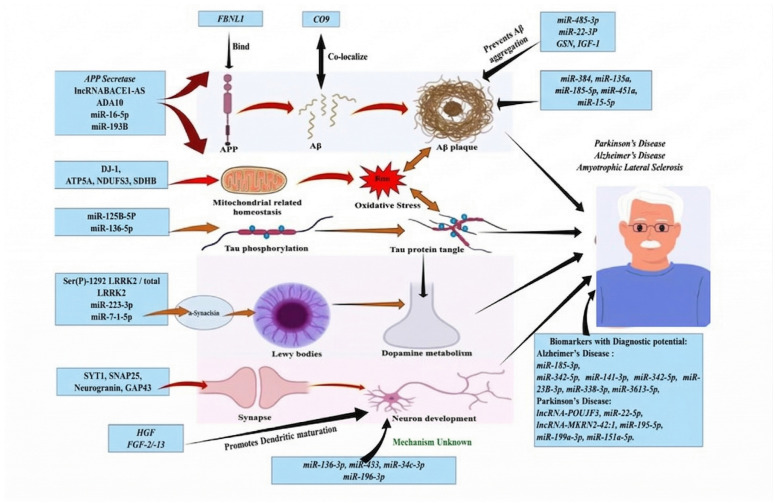
Molecular mechanisms and regulatory networks involved in neurodegenerative diseases. Pathological pathways include BACE1-AS influence on amyloid-beta plaque formation in AD, miRNA regulation of mitochondrial oxidative stress, and tau/alpha-synuclein aggregation. Diagnostic biomarkers for AD and PD are highlighted.

The cell-to-cell transmission of these pathological proteins is actively facilitated by exosomes (extracellular vesicles ranging from 30 to 150 nm), which act as vehicles for disease propagation by carrying toxic proteins like tau, α-synuclein, and mHTT between cells [37,41,42,43,44,45,46,47]. This prion-like spreading relies heavily on the endolysosomal system; when exosomes carrying misfolded tau are taken up by recipient neurons, endolysosomal permeabilization allows toxic tau seeds to escape into the cytosol, initiating widespread intracellular aggregation [43]. In PD, the molecular machinery of the NLRP3 inflammasome actively drives the packaging and transmission of exosomal alpha-synuclein into the extracellular space, exacerbating disease spread [48]. In PD, the molecular machinery of the NLRP3 inflammasome actively drives the packaging and transmission of exosomal alpha-synuclein into the extracellular space, exacerbating disease spread (Figure 2).

#### 2.1.2. Neuroinflammatory Cascades

Chronic neuroinflammation is a shared driver of neuronal death across AD, PD, HD, and ALS, and is meticulously controlled by ncRNA molecular switches [49,50]. Specific miRNAs, such as miR-146a, miR-155, and miR-34a, regulate the inflammatory responses of microglial cells and neurons. At the epigenetic level, lncRNAs like NEAT1, LncTAR, and TUG1 govern glial-mediated inflammatory pathways [25,51] by regulating the activity of chromatin remodeling complexes and localizing them to selected genomic sites to alter inflammatory and stress-related genes [36,52]. This inflammatory landscape is significantly amplified when glial cells secrete exosomes encapsulating key inflammatory regulators like miR-155 and miR-146a; upon transfer to recipient neurons or other glial cells, they trigger cascades that exacerbate neuronal damage [53,54,55]. The pathogenesis of miR-155 in AD is further linked to natural antisense transcripts (NATs), which form highly stable RNA-RNA duplexes to mask microRNA binding sites or dictate mRNA stability [54].

#### 2.1.3. Synaptic Dysfunction and Diagnostic Biomarkers

Beyond proteinopathy and inflammation, dysregulated ncRNAs compromise the brain’s innate regenerative capacity. Aberrant expressions of miR-124 and miR-181 have been directly linked to synaptic dysfunction and progressive cognitive decline [56], while miR-21 and miR-29 control apoptotic pathways dictating neuronal survival or death [40]. Simultaneously, lncRNAs heavily influence neuronal differentiation and synaptic plasticity; for example, HOTAIR and MALAT1 recruit chromatin-modifying enzymes to the Brain-Derived Neurotrophic Factor (BDNF) promoter to influence oligodendrocyte precursor cell differentiation [57], and Meg3 also impacts synaptic plasticity [57,58]. ExosomallncRNAs like XIST and HOTAIR similarly exert significant control over synaptic homeostasis, contributing to the synaptic loss and memory impairments characteristic of AD [59,60]. Exosomal miRNAs can also downregulate crucial neurotrophic factors, such as BDNF, further diminishing neuronal resilience [61,62,63,64].

**Figure 3:**
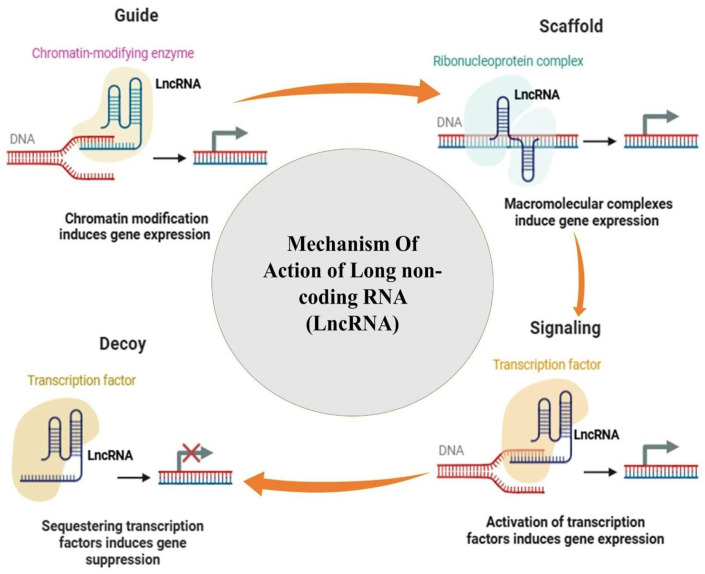
The role of long non-coding RNAs (lncRNAs) in regulating various cellular processes. lncRNAs act as guides for chromatin modifiers, scaffolds for protein-RNA complexes, and decoys for transcription factors to modulate gene expression.

Crucially, because lncRNAs and miRNAs are remarkably stable in biological fluids—often protected from RNase degradation by encapsulation within exosomes or bound to Argonaute proteins—they are emerging as highly promising non-invasive diagnostic and prognostic biomarkers via liquid biopsy [65,66]. Elevated levels of the lncRNA NEAT1 and specific microRNAs like miR-27a-3p in the cerebrospinal fluid (CSF) have been strongly correlated with AD progression and severity, offering a molecular window into the brain before severe cognitive decline manifests. Exosomal miRNA signatures are similarly being profiled to differentiate between PD and atypical parkinsonian syndromes, highlighting their sensitivity as early-stage clinical diagnostic tools. Integrating these transcriptomic profiles into clinical practice will be essential for identifying patients who would benefit most from early RNA-targeted interventions (Figure 3).

### 2.2. Overcoming Barriers: Computational Design and Delivery Engineering

The rational design of RNA therapeutics and their successful delivery across the blood–brain barrier require a synergistic approach between computational modeling and chemical engineering. The rational design of these chemically modified RNA therapeutics relies heavily on advanced computational approaches to analyze massive datasets and identify disease-specific ncRNA alterations [67,68,69,70,71,72]. Bioinformatics platforms, such as TargetScan, miRanda (v3.3a), and RNAhybrid (v2.2), are heavily utilized to predict base-pairing interactions between miRNA sequences and the 3′ untranslated region (UTR) of target mRNAs [73,74,75,76,77,78,79,80,81,82,83,84,85,86,87,88]. To design highly stable and specific therapeutic molecules, algorithms like RNAfold (v2.5.1), Mfold (v3.6), and RNAstructure (v6.4) are employed to model the secondary and tertiary structures of lncRNAs, guiding the targeted binding of small molecules or ASOs to functional RNA domains [71,76,77,89,90,91]. Furthermore, in silico molecular docking software, such as AutoDock (v4.2) and Glide (v9.1), simulates the binding of small molecules to complex ncRNA structures, accelerating the identification of lead compounds capable of disrupting pathological RNA-protein interactions [11,12,13,14,15,16,17,18,19,20,21,22,23,24,25,26,27,28,29,30,31,32,33,34,35,36,37,38,39,40,41,42,43,44,45,46,47,48,49,50,51,52,53,54,55,56,57,58,59,60,61,62,63,64,65,66,67,68,69,70,71,72,73,74,75,76,77,78,79,80,81,82,83,84,85,86,87,88,89,90,91,92,93,94,95,96,97,98,99,100,101,102,103,104,105,106,107,108,109,110,111,112,113,114,115,116,117,118,119,120,121]. Table 1 outlines the key bioinformatics databases, target prediction algorithms, and gene regulatory network models utilized in the current literature to map these crucial interactions and optimize RNA-based therapeutics.

Once these highly specific therapeutic molecules are computationally designed, chemical engineering and nanotechnology must be employed to overcome the formidable delivery barriers of the central nervous system, particularly the blood–brain barrier (BBB).

Despite the clear pathogenic role of ncRNAs, the efficient delivery of RNA-based therapeutics to the brain remains severely restricted by the permeability of the blood–brain barrier (BBB) [94]. To circumvent this, lipid nanoparticles (LNPs) have emerged as the most widely adopted delivery system, engineered to encapsulate RNA molecules, protect them from degradation, and precisely transport miRNAs and lncRNAs to brain cells [95,96,97,98,99,100,101,102]. Polymeric nanoparticles, formed from biocompatible polymers, offer an additional advantage of controlled and sustained RNA release, which is highly beneficial for treating chronic NDs [103,104,105]. Furthermore, engineered exosomes are being actively explored as biomimetic delivery vehicles; due to their inherent ability to cross the BBB, they can be loaded with specific therapeutic RNAs and targeted directly to the brain [95,96].

Naked RNA delivery is therapeutically non-viable without profound chemical engineering to enhance stability, specificity, and cellular uptake [106,107,108,109,110,111]. Chemical modifications, such as the incorporation of 2′-O-methyl groups into the sugar moiety of RNA, protect the molecules from exonuclease degradation and improve pharmacokinetics [112]. Additionally, phosphorothioate modifications, which substitute non-bridging oxygen atoms in the RNA backbone with sulfur atoms, significantly stabilize antisense oligonucleotides (ASOs) targeting lncRNAs or miRNAs [113]. Advanced structural modifications, including Locked Nucleic Acids (LNAs) and Peptide Nucleic Acids (PNAs)—which replace the sugar-phosphate backbone with an amide bond—drastically enhance binding affinity and target specificity, allowing for the precise disruption of disease-associated lncRNAs [113,114,115].

**Figure 4:**
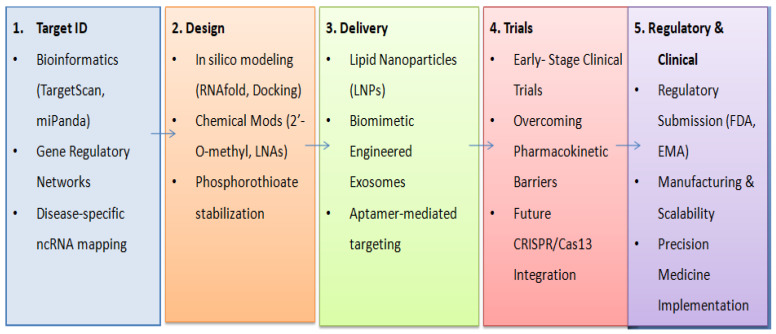
The integrated therapeutic pipeline for non-coding RNAs in neurodegenerative diseases. The process involves a synergistic progression from computational target identification and rational sequence design to chemical stabilization, nanotechnology-driven delivery systems, and eventual evaluation in clinical trials.

Beyond LNPs, the chemical engineering of RNA delivery is advancing rapidly through the use of ionizable lipids and oligonucleotide aptamers. The core chemistry of modern LNPs relies on ionizable cationic lipids that remain neutral at physiological pH—drastically reducing systemic toxicity—but become protonated within the acidic environment of the endosome. This protonation facilitates endosomal escape, releasing the RNA payload directly into the neuronal cytoplasm before lysosomal degradation can occur. To further enhance cellular targeting across the blood–brain barrier, RNA or DNA aptamers are being conjugated to the exterior of these nanocarriers. Aptamers are short, single-stranded oligonucleotides that fold into unique three-dimensional conformations, allowing them to bind to specific neuronal surface receptors with high affinity, functioning much like monoclonal antibodies. This aptamer-mediated targeting drastically improves the specificity of RNA-based therapeutic approaches, ensuring that the ncRNA cargo is delivered exclusively to degenerating neurons or inflamed glia, thereby minimizing off-target effects in healthy brain tissue. The synergistic integration of these computational design and chemical engineering strategies is visualized in Figure 4, illustrating the critical roadmap required for transitioning ncRNA therapeutics from theoretical models to clinical validation.

## 3. Discussion

The transition of ncRNAs from being perceived as transcriptional “junk” to recognized master regulators of gene expression marks a significant paradigm shift in the molecular biology of neurodegenerative diseases [29,115]. While traditional pharmacological interventions predominantly target downstream protein products to manage symptoms, ncRNA-based therapies offer a disease-modifying approach by intervening at the post-transcriptional level [6,122]. This review highlights the immense potential of targeting miRNAs, lncRNAs, and exosomal RNAs to restore cellular homeostasis, reduce neuroinflammation, and mitigate protein aggregation in conditions such as AD and PD [31,33].

### 3.1. Clinical Translation and Mechanistic Insights

The clinical translation of ncRNA therapeutics is currently progressing from preclinical validation to early-stage clinical trials [110,123]. For instance, the inhibition of miR-34a—a known driver of neuroinflammation and tau phosphorylation in AD—is currently under clinical evaluation (NCT04360980) utilizing lipid LNP delivery systems [124,125]. Similarly, targeting the lncRNA BACE1-AS with antisense oligonucleotides (ASOs) has demonstrated preclinical success in reducing amyloid plaque deposition [122,126]. A critical comparison of these targets reveals that while miRNAs exert pleiotropic effects by regulating multiple genes simultaneously [27], lncRNAs offer a higher degree of target specificity, often modulating a singular pathogenic locus or epigenetic complex [32]. This distinction is crucial for minimizing off-target effects during therapeutic design [79,127]. The culmination of these computational and chemical strategies is currently being tested in early-stage clinical trials [123]. For example, preclinical models have shown that inhibiting miR-34a reduces amyloid-beta formation and tau phosphorylation [124]. This has led to the ongoing clinical trial NCT04360980, which evaluates the safety and efficacy of an LNP-delivered miR-34a inhibitor aimed at reversing cognitive decline in AD patients [125]. Similarly, phase I/II trials are being developed for ASOs targeting the BACE1-AS lncRNA to reduce amyloid plaque deposition [122,126,128,129].

CRISPR/Cas technology is emerging as a transformative tool for precisely modulating ncRNA expression [130,131,132,133,134,135,136,137,138]. While CRISPR/Cas9 is traditionally used to create double-strand DNA breaks via guide RNA routing, adapted CRISPR systems are now being investigated to specifically knock down disease-associated lncRNAs or block the production of aberrant miRNAs via catalytically inactive Cas9 (dCas9) fusion proteins [139,140,141,142,143,144]. Direct RNA editing technologies, such as Adenosine-to-Inosine (A-to-I) editing, provide a highly targeted approach to repair malfunctioning RNA molecules without permanently altering the underlying genomic DNA [145,146,147,148]. While challenges regarding off-target effects and efficient brain delivery persist, these precise editing tools establish a new standard in precision medicine, offering the unprecedented potential to halt the progression of neurodegenerative diseases at their molecular roots [127,149,150,151,152,153,154,155,156,157,158,159,160,161].

### 3.2. Challenges and Limitations

Despite the profound therapeutic potential of ncRNA-based interventions, a significant gap remains between robust preclinical findings and successful clinical translation. Several formidable challenges must be addressed before these therapies can achieve widespread clinical utility.

While lipid nanoparticles (LNPs) and exosomes have significantly advanced RNA delivery, efficiently and safely crossing the human blood–brain barrier (BBB) remains the most critical bottleneck in neurotherapeutics. In clinical settings, achieving uniform therapeutic distribution throughout the central nervous system without requiring highly invasive administration routes (such as repeated intrathecal injections) is exceptionally difficult. Furthermore, synthetic nanocarriers like LNPs frequently encounter pharmacokinetic limitations; these include rapid systemic clearance, unintended accumulation in the liver (hepatotoxicity), and the potential to trigger severe immune activation upon repeated dosing. Conversely, while engineered exosomes exhibit superior biocompatibility and natural BBB penetrance, their clinical application is severely hampered by manufacturing and regulatory hurdles. These include a lack of standardized isolation protocols, batch-to-batch structural variability, and profound difficulties in scalable, clinical-grade production.

A fundamental biological limitation of targeting ncRNAs, particularly miRNAs, is their inherent pleiotropy. Because a single miRNA can simultaneously regulate hundreds of downstream mRNA transcripts across various gene networks, therapeutic modulation intended to correct a specific pathological locus may inadvertently disrupt normal physiological pathways in healthy tissues. This lack of singular target specificity leads to unpredictable off-target effects and potential long-term cellular toxicity. This is equally concerning for emerging CRISPR/Cas-based interventions; while highly precise, unintended RNA cleavage or delivery-associated toxicity could induce severe, long-term neurotoxic events if not meticulously controlled.

The current translational landscape is further limited by an over-reliance on transgenic rodent models. While highly informative for basic mechanisms, these animal models often fail to accurately recapitulate the protracted, multifactorial, and age-dependent pathogenesis of human neurodegenerative diseases. Consequently, ncRNA therapies that demonstrate high efficacy and safety in mice frequently yield inconclusive outcomes in human trials. Moving forward, the field must systematically analyze data from both successful and discontinued clinical trials, as unsuccessful outcomes often yield the most critical insights into delivery failures, immune system rejection, and the precise therapeutic windows required to successfully bridge the gap between preclinical promise and real-world clinical applicability.

### 3.3. Future Directions

The next phase of neurodegenerative research must shift from the identification of isolated ncRNA targets toward a comprehensive, multi-modal therapeutic framework that addresses the inherent complexity of brain pathology. A primary recommendation for future study is the development of combination therapies that simultaneously target multiple aspects of disease pathogenesis. Integrating precision ncRNA modulation (e.g., miRNA mimics or specific ASOs) with traditional small-molecule agents or immunotherapies will likely provide more robust disease modification than monotherapies [120,162,163,164,165,166]. This integrated approach is essential because neurodegeneration is rarely driven by a single molecular defect.

Furthermore, the field is moving definitively toward the era of precision medicine [132,133]. By integrating patient-specific genomic and transcriptomic data (RNA-seq) [85,134], it is becoming possible to construct individual disease models to predict therapeutic efficacy and monitor response via circulating exosomal biomarkers [135,136]. Personalized ncRNA profiles will allow for the selection of patient-specific interventions, maximizing efficacy while minimizing off-target toxicity. Computational modeling and Artificial Intelligence (AI) will play an increasingly dominant role in this process, providing the predictive power needed to simulate the behavior of these therapies and their interactions within complex gene regulatory networks.

To realize this potential, the advancement of delivery technologies remains a critical priority. Future research should prioritize the engineering of “Exosome 2.0” platforms—natural extracellular vesicles modified with specific peptides or antibodies to achieve high-precision targeting of afflicted cell types, such as dopaminergic neurons in Parkinson’s disease or hippocampal neurons in Alzheimer’s disease. Simultaneously, the refinement of lipid and polymeric nanoparticles must focus on creating biodegradable systems that prevent long-term tissue accumulation and potential inflammatory side effects.

Finally, emerging technologies such as CRISPR/Cas13 and precise RNA base-editing represent the next frontier in neurotherapeutics [137,147]. Unlike Cas9, which permanently alters DNA and carries significant ethical and long-term safety concerns [139,146], RNA-centric CRISPR systems allow for the transient, highly specific knockdown of dysregulated lncRNAs or miRNAs [141,148]. However, challenges remain regarding the delivery of these large CRISPR components to the brain and the long-term maintenance of therapeutic effects [150]. Future research must focus on optimizing these delivery vectors. By integrating these diverse disciplines—bioinformatics, chemical engineering, and precision molecular biology—the next decade of research holds the potential to transition from managing the symptoms of neurodegeneration to successfully halting its molecular progression.

## 4. Conclusions

The exploration of non-coding RNAs marks a critical paradigm shift in neurodegenerative disease research, moving beyond palliative symptom management to directly target the upstream molecular origins of pathology. While biological barriers such as RNA instability and the blood–brain barrier remain formidable, the strategic convergence of advanced computational modeling, chemical engineering, and nanotechnology is systematically dismantling these obstacles. By leveraging AI-driven target prediction, stabilized RNA chemistries, and biomimetic delivery systems like engineered exosomes, the precise modulation of dysregulated ncRNA networks is becoming clinically viable. Ultimately, as the field integrates emerging CRISPR/Cas13 editing tools and personalized transcriptomic profiling, RNA-based therapeutics are poised to offer a transformative, disease-modifying framework for currently untreatable neurodegenerative conditions.

## Figures and Tables

**Table 1:** Distribution of computational tools and bioinformatics databases utilized in the reviewed literature, including the corresponding reference citations and the total number of articles cited for each resource.

Bioinformatics Database/Tool	Version/URL (Access Date)	Reference Number(s) Cited	Total Articles Cited
ncRNADisease	v4.0/http://www.rnadisease.org (accessed on 1 January 2026)	[83]	1
RNAcentral	https://rnacentral.org (accessed on 1 January 2026)	[70]	1
PicTar	https://pictar.mdc-berlin.de (accessed on 1 January 2026)	[70]	1
GeneMANIA	https://genemania.org (accessed on 1 January 2026)	[84]	1
Cytoscape	v3.10/https://cytoscape.org (accessed on 1 January 2026)	[74]	1
RegNetwork	http://www.regnetworkweb.org (accessed on 1 January 2026)	[84]	1
IRegulon	v1.3 (Cytoscape App)/http://iregulon.aertslab.org (accessed on 1 January 2026)	[84]	1
ORegAnno	v3.0/https://www.oreganno.org (accessed on 1 January 2026)	[84]	1
Enrichr	https://maayanlab.cloud/Enrichr (accessed on 1 January 2026)	[85]	1
DeepMinD	https://github.com/xypan1232/DeepMinD (accessed on 1 January 2026)	[85]	1
DeepTarget	https://github.com/midas-research/deeptarget (accessed on 1 January 2026)	[85]	1
TargetScan	Release 8.0/http://www.targetscan.org	[86]	1
miRanda	v3.3a/http://cbio.mskcc.org/microrna_data/manual.html (accessed on 1 January 2026)	[86]	1
LncBase	v3.0/https://dianalab.e-ce.uth.gr/lncbasev3	[87]	1
LncRNAdb	http://www.lncrnadb.org (accessed on 1 January 2026)	[87]	1
RNAhybrid	https://bibiserv.cebitec.uni-bielefeld.de/rnahybrid (accessed on 1 January 2026)	[88]	1
ExoRBase	v2.0/http://www.exorbase.org (accessed on 1 January 2026)	[88]	1
ExoCarta	Version 5/http://www.exocarta.org	[88]	1
BindingDB	https://www.bindingdb.org (accessed on 1 January 2026)	[80]	1
ChEMBL catalogue	Release 33/https://www.ebi.ac.uk/chembl (accessed on 1 January 2026)	[87]	1

## Data Availability

No new data were created or analyzed in this study. Data sharing is not applicable to this article.
